# A computationally efficient algorithm for fitting ion channel parameters

**DOI:** 10.1016/j.mex.2016.11.001

**Published:** 2016-11-16

**Authors:** Zachary R. Teed, Jonathan R. Silva

**Affiliations:** Department of Biomedical Engineering, Washington University, St Louis, MO, United States

**Keywords:** The matrix exponential method for ion channel parameterization, Cardiac action potential, Ion channels, Computer modeling

## Abstract

Continuous time Markov models have been widely used to describe ion channel kinetics, providing explicit representation of channel states and transitions. Fitting models to experimental data remains a computationally demanding task largely due to the high cost of model evaluation. Here, we propose a method to efficiently optimize model parameters and structure. Voltage clamp channel protocols can be decomposed into a series of fixed steps of constant voltage resulting in a set of linear systems of differential equations. Given the linear systems, ODE integration can be swapped for the faster matrix exponential routine. With our parallelized implementation, optimized models are able to reproduce a wide range of experimentally collected data within one minute, a 50 times speedup over ODE integration.

• The cost of the objective function is reduced by employing the matrix exponential
• The likelihood of convergence is improved by applying synchronous start simulated annealing
• The approach was tested by optimizing parameters for a model of the cardiac voltage-gated Na^+^ channel, Na_V_1.5, and the KCNQ1 K^+^ channel.

## Graphical abstract

**Figure:**
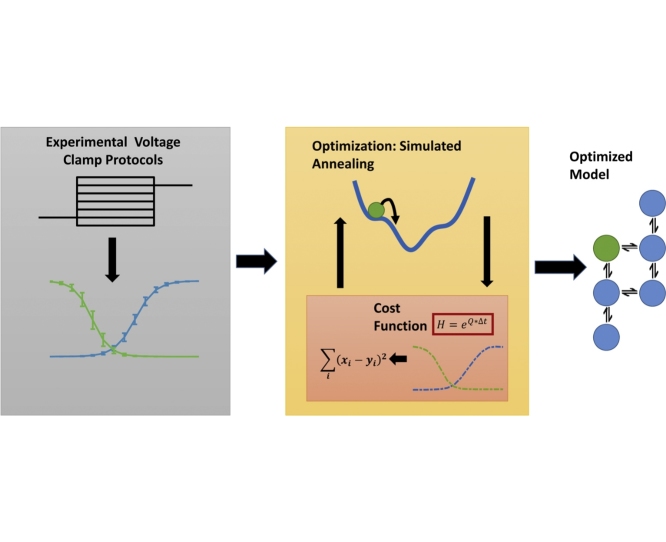

## Method

Following the seminal work of Hodgkin and Huxley in 1952 [1], mechanistic ordinary differential equation (ODE) models have been used to simulate dynamics of excitable systems including neurons, myocytes and pancreatic beta cells [2], [3]. A persistent challenge in creating these models is the identification of optimal parameters. This difficulty arises from the multi-dimensionality of the search, the exponential form of the rate equations, and the existence of many local minima. Global search methods such as simulated annealing and genetic algorithms have been applied to identify optimal sets of parameters [4], [5], [6]. However, these approaches require significant computational resources.

The primary bottleneck for optimization is the numerical solution of ODEs needed to simulate experimentally observed dynamics. When evaluating a large set of models with global searches, this step becomes rate-limiting, and is particularly difficult when the equations become stiff, requiring very small time-steps or complicated implicit schemes [7].

**Fig. 1:**
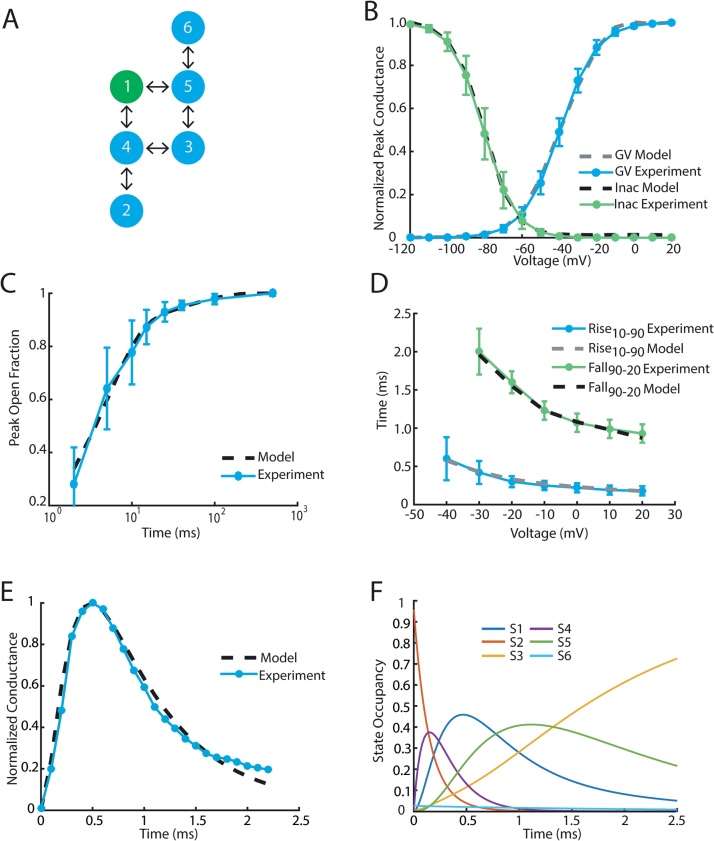
Overview of Na^+^ model fitting results. (A) Architecture of the fitted model, open state is shown in green. (B) Voltage dependent activation (G-V) and steady state inactivation (SSI) model simulations compared with experiment using protocols as described in reference [27]. (C) Recovery from single depolarizing pulse model simulation compared with experiment. (D) Rise time (10–90%) and fall time (90–20%) of normalized conductance from model and experiment. (E) Normalized conductance trace −10 mV depolarizing pulse. (F) Simulated state occupancy from a −10 mV pulse. According to the model, a depolarizing voltage pulse causes the channel to transition from the closed state (S2) through S4 to the conducting state (S1). The channel inactivates by transitioning from the conducting through S5 to the inactive state (S3).

**Fig. 2:**
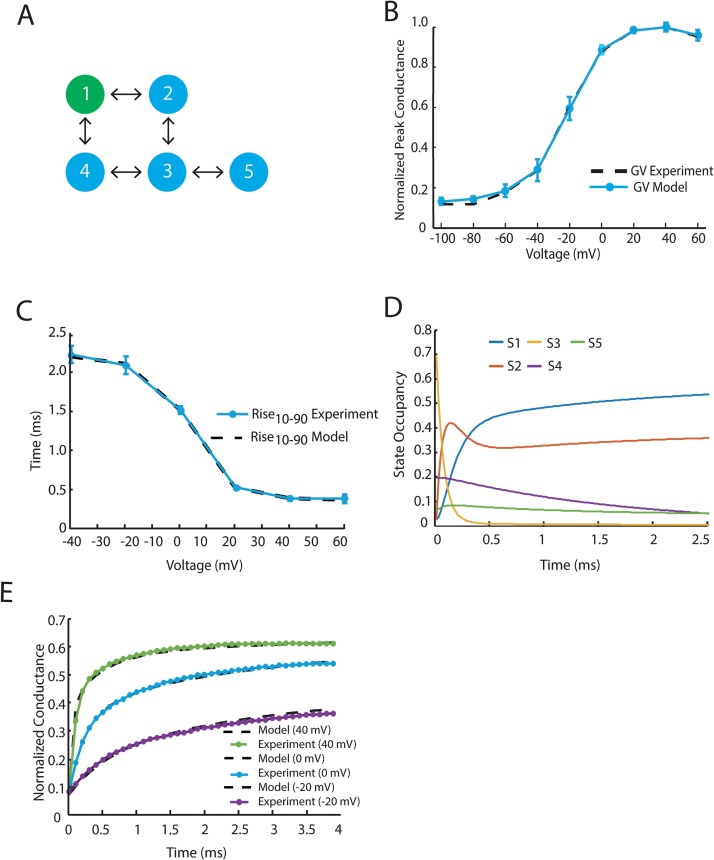
Overview of K^+^ model fitting results. (A) Architecture of the fitted model, open state is shown in green. (B) Voltage dependent activation model simulations compared with experiment. (C) Deactivation simulation compared to experiment (D) Rise time (10–90%) of conductance from model and experiment. (E) Normalized conductance trace of −20 mV, 0 mV, and 40 mV depolarizing pulses from a resting potential of −80 mV. (F) Simulated state occupancy from a −10 mV pulse. When exposed to a depolarizing pulse, the model transitions from the closed state (S3) through S4 to the open state (S1). (For interpretation of the references to color in this figure legend, the reader is referred to the web version of this article.)

We propose to overcome the ODE barrier by solving these differential equations with the matrix exponential, an approach that has been used to analyze channel kinetics and improve the efficiency of action potential simulations [8], [9]. We tested this approach on experimental voltage pulse protocols, optimizing a two discrete-state Markov models of the cardiac Na^+^ channel and the KCNQ1 K^+^ channel. Optimization was performed using multiple chain simulated annealing [10]. Code has been made publically available at, where demos and https://github.com/silvalab/MMOptimizer instructions on model fitting can be found. Example model graphs and fits to Na^+^ experimental data are shown in Fig. 1; K^+^ fits are shown in Fig. 2.

We used the following voltage pulse protocols for Na^+^ model fitting:

*Voltage Dependent Activation* (Fig. 1B)*:* A series of depolarizing pulses were applied from −120 mV to 20 mV in 10 mV increments from a resting potential of −100 mV. The voltage-dependence of channel conductance was found from this protocol by finding the peak current during the pulse and dividing by the driving force ($V_{m}-E_{Na}$), where $V_{m}$ is the membrane potential and $E_{Na}$ is the reversal potential for Na^+^.

*Steady State Inactivation* (Fig. 1B)*:* Cells were held at −120 mV before being exposed to conditioning pulses ranging from −120 mV to 20 mV in 10 mV increments for 200 ms. Voltage was then stepped to −20 mV and peak current was recorded and normalized by dividing by the maximum current.

*Recovery from Inactivation* (Fig. 1C)*:* Cells were held at −120 mV before being exposed to a −20 mV depolarizing pulse for 200 ms. Cells were then allowed to recover at −120 mV for durations ranging from 1 ms to 1000 ms before a final depolarizing pulse at −20 mV where peak current was recorded and normalized across samples.

*Rise/Fall Time* (Fig. 1D)*:* This protocol replicated *Voltage Dependent Activation*. Rise (10%-90%) measured the amount of time it took for current to rise from 10% to 90% of the maximum recorded current. Fall (90%-20%) characterized fast inactivation kinetics by measuring the time it took for current to fall from 90% to 20% peak current.

*Conductance Trace* (Fig. 1E): In the final protocol we exposed the cell to a single depolarizing pulse and recorded normalized conductance over 5 ms.

Similarly, we used the following voltage pulse protocols for K^+^ model fitting:

*Voltage dependent activation* (Fig. 2B): A series of depolarizing pulses were applied from −100 mV to 60 mV in 20 mV increments from a resting potential of −100 mV. The voltage-dependent channel conductance was calculated by dividing the peak current by the driving force ($V_{m}-E_{K}$), where $V_{m}$ is the membrane potential and $E_{K}$ is the reversal potential for K^+^.

*Deactivation* (Fig. 2C): Cells were held at −80 mV before exposure to a depolarizing pulse at 60 mV for 2 ms. Voltage was then stepped to conditioning pulses ranging from −120 mV to 0 mV for 4 ms. Voltage dependent-channel conductance was calculated by dividing the minimum absolute current by the driving force.

*Rise Time* (Fig. 2D)*:* This protocol replicated *Voltage Dependent Activation*. Rise (10%-90%) measured the amount of time it took for the current to rise from 10% to 90% of the peak.

*Conductance Traces* (Fig. 2E): Cells were exposed to −20 mV, 0 mV, and 40 mV depolarizing pulses and normalized conductance was recorded over a 4 ms interval.

## Model structure

Models are implemented as a connected graph, a sequence of rate parameters, and a boolean conductivity vector. Each node in the graph represents a discrete model state, with transitions between model states denoted by graph edges. The conductivity vector gives the relative conductance for each state. Models are initialized from the space of random connected graphs using an approach described by Wilson et al. [11]. We begin by constructing a minimum spanning tree by performing a random walk over the graph until all nodes have been visited. Every time an unvisited node is encountered, the edge connecting this node to the previous node is added to the graph. Afterwards, new edges are added with constant probability. Initial rate parameters are drawn from independent normal distributions.

If we let e∈E denote the set of edges and r∈R the set of rates, the transition matrix Q can be generated as follows:

(1) $$\begin{array}{c} Q_{ij}=\{\begin{array}{c} r_{ij},\left(i,j\right)\varepsilon E\\ 0,otherwise \end{array}fori\neq j \end{array}$$

(2) $$\begin{array}{c} Q_{ii}=-\underset{\left(i,k\right)\varepsilon E}{\sum }r_{ik} \end{array}$$

Note that each edge is bidirectional, so (i,j)εE⇔(j,i)εE. With the transition matrix, the model kinetics can be defined by a single differential equation

(3) $$\begin{array}{c} \frac{dx}{dt}=Qx\;\;\;\;\; \end{array}$$

where $\vec{x}$ is the vector of state occupancies. The solution to this ODE can be approximated by using standard solvers such as Runge-Kutta methods [12].

If we limit ourselves to the unconstrained case, we can optimize over all r∈R rate parameters. However, most ion channels are observed to obey microscopic reversibility, or the reversibility of flow in closed loops [13], [14], so we instead limit the search space to the set of models that satisfy this principle. Rather than attempting to solve the constrained optimization problem, we follow the approach outlined by Menon et al. [4], where we optimize over a set of independent rate parameters which can be used to generate models that observe microscopic reversibility.

Microscopic reversibility requires that in equilibrium, the transition rates between any two connected states must balance each other [15]. Thus, if (i,j)∈E, the following must hold:

(4) $$\begin{array}{c} r_{ij}s_{i}=r_{ji}s_{j}\;\;\;\;\;\;\;\;\;\;\;\;\;\; \end{array}$$

where $s_{i}$ and $s_{j}$ are the steady state occupancies for states i and j respectively. Taking the log of both sides, Eq. (4) can be written in matrix form

(5) $$\begin{array}{c} \ln\left(r_{ij}\right)-\ln\left(r_{ji}\right)=\ln\left(s_{j}\right)-\ln\left(s_{i}\right)\;\;\;\;\;\; \end{array}$$

(6) $$\begin{array}{c} Dln\left(r\right)=I_{e}^{\text{T}}\ln\left(s\right)\;\;\;\;\; \end{array}$$

where $I_{e}^{T}$ is the transpose of the even columns of the graph’s incidence and D is a E × 2E difference matrix with

(7) $$\begin{array}{c} D_{ij}=\{\begin{array}{c} 1,j=2i\\ -1,j=2i+1\\ 0,otherwise \end{array}\;\;\;\;\; \end{array}$$

This forms a system of equations with 2E unknowns and E equations. To solve for R, we need an additional E independent equations. A simple choice is to define a new variable k to be the product of forward and backward rates

(8) $$\begin{array}{c} k_{ij}=r_{ij}r_{ji}\;\;\;\;\;\;\;\;\;\;\; \end{array}$$

(9) $$\begin{array}{c} \ln\left(k_{ij}\right)=\ln\left(r_{ij}\right)+\ln\left(r_{ji}\right)\;\;\;\;\; \end{array}$$

or again written in matrix form

(10) $$\begin{array}{c} abs\left(D\right)\ln\left(r\right)=\ln\left(k\right)\;\;\;\; \end{array}$$

Combining Eq. (9) with Eq. (6) gives an independent linear systems of equations with 2E variables and 2E equations.

(11) $$\begin{array}{c} \left[\begin{array}{c} D\\ abs\left(D\right) \end{array}\right]\ln\left(r\right)=\left[\begin{array}{c} I_{e}^{T}\ln\left(s\right)\\ \ln\left(k\right) \end{array}\right]\;\;\;\; \end{array}$$

(12) $$\begin{array}{c} \ln\left(r\right)={\left[\begin{array}{c} D\\ abs\left(D\right) \end{array}\right]}^{-1}\left[\begin{array}{c} I_{e}^{T}\ln\left(s\right)\\ \ln\left(k\right) \end{array}\right]\;\;\;\;\;\; \end{array}$$

Of course, we expect the elements of the rate vector to be voltage-dependent, but microscopic reversibility must also hold for all values of voltage. Eq. (12) shows that ln(r) is linear in terms of ln(s) and ln(k). Thus, we can define s(v) and k(v) to the exponent of a linear operation

(13) $$\begin{array}{c} s_{i}\left(v\right)=\exp\left(f\left(v\right)\cdot \alpha_{i}\right)\;\;\;\; \end{array}$$

(14) $$\begin{array}{c} k_{ij}\left(v\right)=\exp\left(f\left(v\right)\cdot \beta_{\mathrm{ij}}\right)\;\;\;\; \end{array}$$

where f(v) is a vector valued function of voltage, and α,β are vectors of rate parameters which are subject to optimization. Substituting Eqs. (13) and (14) into Eq. (12)

(15) $$\ln(r)={\left[\begin{array}{c} D\\ abs(D) \end{array}\right]}^{-1}[\begin{array}{c} {\begin{array}{c} I \end{array}}_{e}^{T}\alpha f{\left(v\right)}^{T}\\ \beta f{\left(v\right)}^{r} \end{array}]={\left[\begin{array}{c} D\\ abs(D) \end{array}\right]}^{-1}{\left[\begin{array}{c} {\begin{array}{c} I \end{array}}_{e}^{T}\alpha \\ \beta \end{array}\right]}^{-1}f{\left(v\right)}^{T}$$

Substituting the voltage constant part of Eq. (15) for θ

(16) $$\begin{array}{c} \theta ={\left[\begin{array}{c} D\\ abs\left(D\right) \end{array}\right]}^{-1}\left[\begin{array}{c} I_{e}^{T}\alpha \\ \beta \end{array}\right]\;\;\;\;\;\; \end{array}$$

(17) $$\begin{array}{c} r\left(v\right)=\exp\left(\theta \cdot f{\left(v\right)}^{T}\right)\;\;\;\; \end{array}$$

The results obtained in Eq. (17) can be used to generate the transition matrix as shown in Eq. (2). Because the rate vector is the result of linear operations on ***s*** and ***k*** it is of the same form as ***s*** and ***k***.

## Voltage dependence

In the model, transition rates are given as the exponent of the inner product of a vector valued voltage function and the model parameters.

(18) $$\begin{array}{c} r\left(v\right)=e^{f\left(v\right)\cdot \theta }\;\;\;\; \end{array}$$

Any rate function that can be written in this form can be made to satisfy microscopic reversibility by the method previously described. The simplest voltage function is a linear model motivated by the constant temperature Eyring rate theory [16], with $f\left(v\right)=\left[\begin{array}{cc} 1 & v \end{array}\right]$. The transition rate then becomes

(19) $$\begin{array}{c} r\left(v\right)=e^{\theta_{1}+v\theta_{2}}\;\;\;\; \end{array}$$

In practice, the linear model worked well. We were able to obtain good fits for all provided voltage clamp protocols. Nonetheless, the linear model is prone to stiff differential equations. As membrane voltage becomes increasingly large (for $\theta_{2}>0$) or increasingly small (for $\theta_{1}<0$), the r(v) becomes exponentially larger, often resulting in physically implausible transition rate. A simple solution would be to bound r(v); however, in models where the rates are not linearly independent microscopic reversibility could be violated.

Another proposed voltage function extends the linear model by adding higher order polynomial terms [17], with $f_{i}\left(v\right)=v^{i-1}$ and $r\left(v\right)=e^{\theta_{1}+\theta_{2}v+\ldots +\theta_{n}v^{n-1}}$. We test both second order expansions of this form. The higher order terms gave the model more expressive power and resulted in significantly reduced fitting error. Regardless, the polynomial model showed signs of severe overfitting. Even slight modifications of the protocol parameters resulted in very different behavior, making models ill-suited for larger scale simulations.

Finally, we attempted a third voltage dependence function in attempt to circumvent some of the limitations of the linear model by bounding transitions rates while not violating microscopic reversibility. The proposed function is in the form

(20) $$\begin{array}{c} f\left(v\right)=\left[\begin{array}{cc} 1 & sig(\frac{v-a}{b} \end{array})\right]\;\;\;\;\; \end{array}$$

where sig is the sigmoid function and a and b are fixed parameters shared across all rates. In regions where v≈a, the sigmoid model closely approximates the linear model; however, at large or small voltages, the sigmoid function becomes bounded limiting the maximum rate and reducing ODE stiffness. Additionally, this function provided better fits than the linear model.

## Cost function evaluation

**Fig. 3:**
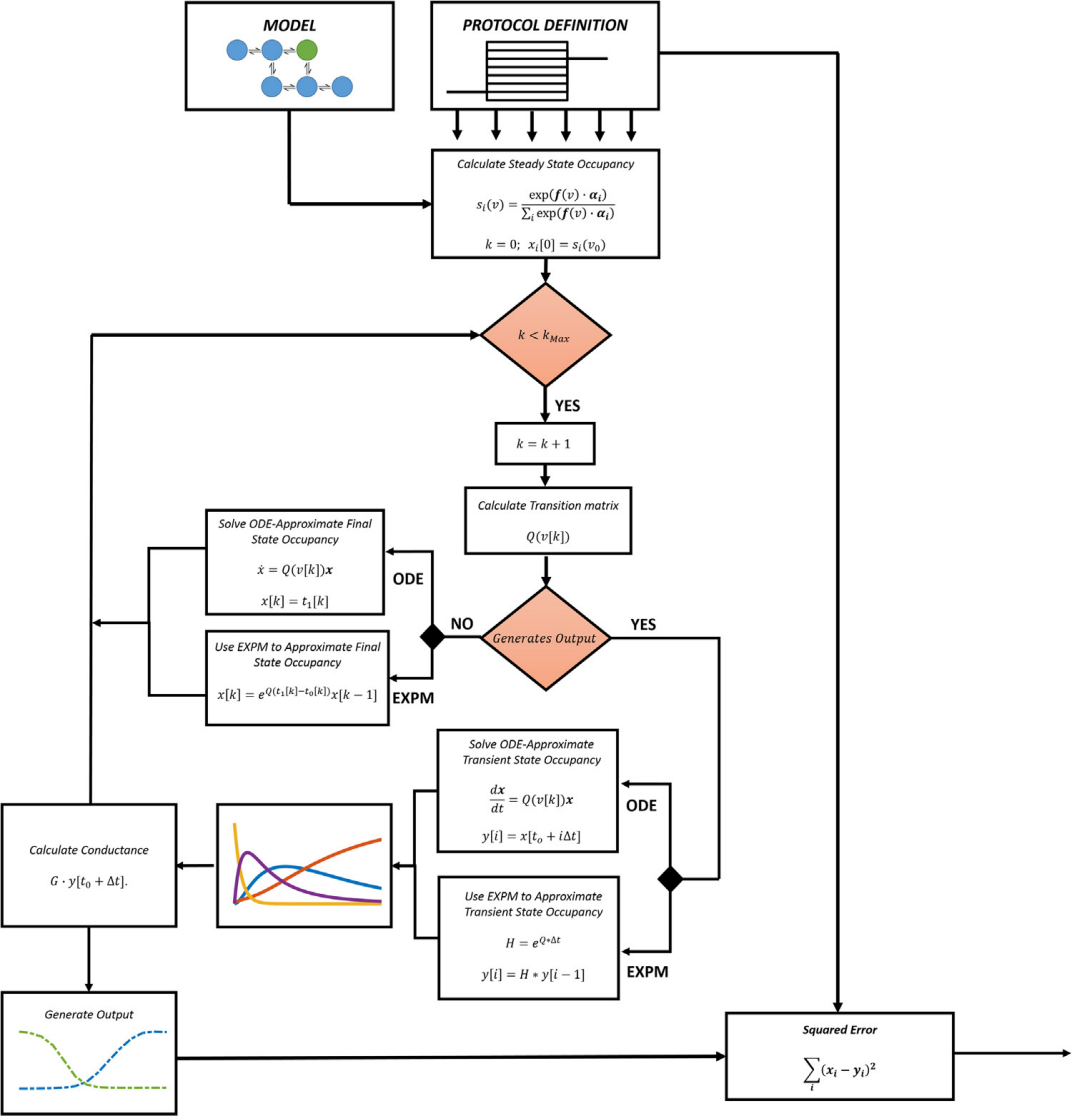
Cost function evaluation using matrix exponential/ODE solver. The cost function takes the model and protocol definition as input and outputs the squared error between the model and experimental outputs. Detailed information and instructions regarding protocol encoding can be found https://github.com/silvalab/MMOptimizer. The main procedure is repeated for each voltage trace in each protocol. The error for each trace is computed by comparing the simulation outputs to experimentally collected values; whether or not a step produces any output is encoded in the protocol design. First, the steady state occupancy is computed for the resting membrane potential. Next, we iterate through each step in the voltage clamp protocol, beginning at $t_{0}\left[k\right]$ and ending with $t_{1}\left[k\right]$ with step voltage denoted by v[k]. Q(v[k]) is calculated using Eq (2). If the current step does not produce output, we simply compute the state occupancy x[k] at $t_{1}\left[k\right]$. Using ODE integrators we can approximate x[k] by solving $\dot{x}=Q\left(v\left[k\right]\right)x$ with initial state x(0)=x[k−1]. Using the matrix exponential we simply compute $x\left[k\right]=e^{Q\left(t_{1}\left[k\right]-t_{0}\left[k\right]\right)}x\left[k-1\right]$. On the other hand, if the step does produce output, we are forced to use a small step size Δt to sample state occupancy over the range $t_{0}\left[k\right]$ to $t_{1}\left[k\right]$. Using the ODE solver, intermediate state occupancy is recorded for each time step for all $is.t.i\Delta t<t_{1}-t_{0}$. When using the matrix exponential, *H* is precomputed as $H=e^{Q*\Delta t}$. Each step is then computed by ‘powering up’ the matrix exponential y[i]=H⋅y[i−1]. Both methods then share the next step in which normalized conductivity is computed by performing the dot product of the state occupancy matrix y with the conductivity vector G. This giving the normalized channel conductivity over the timespan $t_{0}\left[k\right]$ to $t_{1}\left[k\right]$, providing the raw data necessary to output derived features such as peak conductance, rate constants, and normalized traces.

Each of the fitted voltage clamp protocols consist of a sequence of discrete voltage steps. The cost function is computed by simulating each of the protocols, recording the results, and using squared error as a metric to evaluate model performance. In this paper, we look at two different ways to simulate the model: traditional ODE solvers and the matrix exponential. A flow chart illustrating the cost function evaluation for both approaches is show in Fig. 3.

**Fig. 4:**
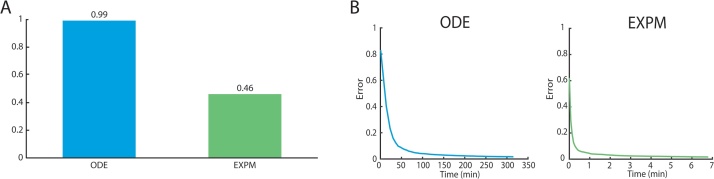
(A) Execution time profile of model fitting. When using ode solvers, channel simulation accounts for 99% of execution time, compared with 46% in the EXPM implementation. (B) Average model error as a function of execution time for both EXPM and ODE implementations using a single process. Error converges in approximately 300 min when using ode integration; this time is reduced to five minutes with matrix exponentiation. These results agree with [23] where ODE methods were shown to take significantly longer and obtain lower overall accuracy.

As the execution time profiling results in Fig. 4A show, ODE integration is the clear bottleneck, accounting for as much as 99% of execution time. ODE solvers were designed to solve a broad class of problems

(21) $$\begin{array}{c} \dot{x}=f\left(x,t\right),x\left(0\right)=x_{0}\;\;\;\;\; \end{array}$$

Yet, voltage clamp protocols commonly consist of a series of discrete voltage steps, reducing the system from $\dot{x}=Q\left(v\left(t\right)\right)x$ to$\dot{x}=Q\left(v\right)x$ where Q(v) is constant over the time $t_{0}\left[k\right]$ to $t_{1}\left[k\right]$. We expect that standard ODE solvers are not able to take full advantage of the linear nature of the constant voltage steps. In contrast, using the matrix exponential, the solution is given as $x\left(t\right)=e^{tQ}x_{0}$ [23]. The matrix exponential can further be used to sample state occupancy over a series of fixed time steps

(22) $$\begin{array}{c} x\left(t+n\Delta t\right)=Q^{n}x\left(t\right)=Qx\left(t+\left(n-1\right)\Delta t\right)\;\;\;\; \end{array}$$

showing that it can effectively replace ODE solvers in our cost function evaluation routine. In our implementation we used ExpoKit’s Padé approximation routine [19], [23]

The resulting speedup is substantial—using a single thread on an Intel Xeon E5-2630 processor, the matrix exponential (EXPM) implementation converges in significantly less time than ode integration (ODE) as shown in Fig. 4B. With a single thread, the EXPM implementation was able fit models in the order of five minutes compared to five hours, a speed-up of more than 50×. Synchronous chain simulated annealing can easily be modified to use more than a single thread. Using 12 threads, model fitting shown in Fig. 1 can be performed in under a minute.

**Fig. 5:**
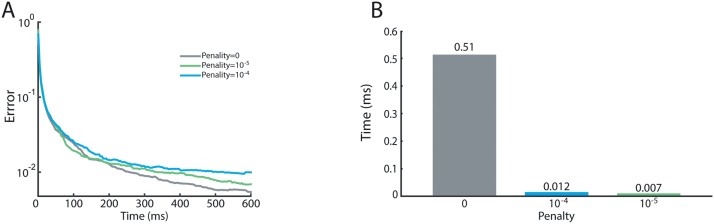
(A) Impact of stiffness penalty on fitting error. The stiffness penalty imposes an additional cost on models proportional to the largest eigenvalue of the rate matrix for a range of voltages. (B) Impact of stiffness penalty on ODE integration time. Following the experiment shown in C, the fitted models were again run over the protocols except using an ODE solver. The values show the average per model execution time.

Finally, we evaluated a stiffness penalty on the models proportional to the model’s largest eigenvalue over a range of voltages (–120 mV to +20 mV). This penalty constrains the search space and resulting models have slightly higher cost (Fig. 5A). Nonetheless, adding a small penalty proved to be important. Following optimization, we reran the fitted models over the protocols, using ODE solvers rather than the matrix exponential (Fig. 5B). Models with no added penalty took over 50 x longer due to their stiff differential equations. In whole cell simulations, membrane voltage is no longer a series of discrete steps, but rather a continuous variable requiring ODE integration. This penalty ensures that fitted models can be efficiently used in larger scale simulations, while constraining transition rates to physically plausible values.

## Optimization

Similar to Menon et al., we optimize over model architecture as well rate parameters, maintaining microscopic reversibility by encoding rates as a set of independent parameters. Instead of a genetic algorithm, we use simulated annealing to explore the combinatorial search space. Genetic algorithms involve a recombination step, where individuals are selected to form offspring for the next generation [22]. With like structured models, new model rates can simply be drawn from a random distribution parametrized by the parents’ rates. When models have different structures (i.e. different numbers of nodes and connectivity), recombination is less obvious. One option is to only perform recombination over models with the same structure. However, we observed this leads to a collapsed structural search space, and leads to unsatisfactory local optima. We obtained better convergence results in much less time using simulated annealing.

Specifically, we used synchronous chain simulated annealing for model optimization, considering the tradeoff between execution time and model error. Simulated annealing is an iterative stochastic search algorithm which combines local Monte Carlo search with an annealing schedule [10]. It probabilistically guarantees a globally optimal solution provided an infinitely long cooling schedule [20]; however, in practice, we wish to fit good suboptimal models quickly.

At each iteration we generate a new model proposal by perturbing the current model $m^{\prime }∼m$**.**

Perturbations are reversible and can alter both model structure and rate parameters taking one or more of the following forms:

1. State Addition: A new state is added to the model. A random edge is formed connecting the new state to a random state drawn from the existing model. The new rate parameters governing the edge and state are drawn from a normal distribution.
2. State Removal: A state is randomly chosen from the existing model and removed; all edges connecting this state are also removed. If removal results in a disconnected graph, the graph is reconnected by forming a random minimum spanning tree over the disconnected components.
3. Edge Addition: Two model states are randomly selected, if these states are not already connected a new edge is formed. Rate parameters are drawn from a normal distribution.
4. Edge Removal: A random model edge is selected and removed. If removal results in a disconnected graph, the graph is reconnected similar to (2).
5. Rate Perturbation: Each rate parameter has probability p of being updated. Updates are performed by adding a random variable drawn from a zero mean normal distribution to the current parameter. Thus, for rate parameter $\theta_{i}$

(23) $$\begin{array}{c} \theta_{i}=\theta_{i}+x_{i}y_{i}\;\;\;\;where\;\;\;\;\;x_{i}∼Ber\left(p\right),y_{i}∼N\left(0,\sigma \right)\;\;\;\; \end{array}$$

The locality of the perturbation can be modified by adjusting hyper-parameters p and σ. Every perturbation performs a rate update—other structural modifications are performed with a predefined probability.

Following perturbation, the cost function for the new model is computed. The new model is accepted with probability

(24) $$\begin{array}{c} P\left(e,e^{\prime },T\right)=\max\left(\exp\left(-\frac{e^{\prime }-e}{T}\right),1\right)\;\;\;\;\; \end{array}$$

where e is the previous model error, e' is the perturbed model error, and T the annealing temperature [21]. As the temperature decreases, T→0, the probability of making an uphill transition, $e^{\prime }>e$, becomes increasingly small. In our implementation, we adopt the simple exponential annealing schedule [21] with $T\left(t\right)=T_{0}\gamma^{t}$ or equivalently T(t)=T(t−1)γ, where γ governs the rate of annealing and initial temperature $T_{0}$.

To avoid getting trapped in local optima and increase the probability of convergence, we run multiple synchronous chains [10]. Rather than a single model, we maintain a set of models, each with their own search path. Model perturbation and acceptance are both performed individually for each chain.

The optimization procedure attempts to fit all the voltage clamp protocols while minimizing model complexity. We found that in many cases, several models of similar complexity were able to achieve approximately the same fitting error. Hence, different runs often converged to different model structures. Since model structure is an optimization variable, we make no attempt to distinguish the types of channel states, rather such states naturally emerge as a product of the optimization. Open states can easily be identified by examine the conductivity vector. Similarly, closed and inactivated states can be identified by exposing models to a depolarizing test pulse (Fig. 1F), and looking at the transition of state occupancy.

## Conclusions

Identification of continuous Markov model parameters is a challenging problem due to a large search space and a high cost of function evaluation, often requiring significant computational resources. We propose to overcome these limitations by efficiently encoding the cost function as a sequence of discrete voltage steps, solvable using the matrix exponential. Our implementation successfully captures channel kinetics without requiring excess resources or time.

Overall, we observed a 50× speedup when using the matrix exponential implementation of the cost function. One a single processor, optimization could be performed in only 10 min—extended to 12 cores, convergence time dropped to under a 2 min. The encoding of the cost function can be generalized to other voltage clamp protocols, allowing our implementation to be easily adapted to fit other channels types and data.

Modern biophysics methods are beginning to reveal molecular details of channel function [24], [25], [26], [27], the nanoscale consequences of inherited mutations [27], [28], and the precise mechanisms of small-molecule drug regulation [29]. To rigorously describe these molecular details, the complexity of models and the burden of optimization will increase substantially, necessitating advanced optimization methods and computer hardware. As such we expect that much future work will be needed to develop the methods required to create these models.

The matrix exponential method for ion channel parameterization
